# Stoichiometric Constraints Do Not Limit Successful Invaders: Zebra Mussels in Swedish Lakes

**DOI:** 10.1371/journal.pone.0005345

**Published:** 2009-04-29

**Authors:** Rahmat Naddafi, Peter Eklöv, Kurt Pettersson

**Affiliations:** 1 Department of Ecology and Evolution/Erken Laboratory, Evolutionary Biology Centre, Uppsala University, Norrtälje, Sweden; 2 Department of Ecology and Evolution/Limnology, Evolutionary Biology Centre, Uppsala University, Uppsala, Sweden; University of Zurich, Switzerland

## Abstract

**Background:**

Elemental imbalances of carbon (C): nitrogen (N): phosphorus (P) ratios in food resources can constrain the growth of grazers owning to tight coupling between growth rate, RNA allocation and biomass P content in animals. Testing for stoichiometric constraints among invasive species is a novel challenge in invasion ecology to unravel how a successful invader tackles ecological barriers in novel ecosystems.

**Methodology/Principal Findings:**

We examined the C∶P and N∶P ratios and the condition factor of a successful invader in lakes, the zebra mussel (*Dreissena polymorpha*), collected from two Swedish lakes. Concurrently, we analyzed the elemental composition of the food (seston) and tissue of the mussels in which nutrient composition of food and mussels varied over time. Zebra mussel condition factor was weakly related to the their own tissue N∶P and C∶P ratios, although the relation with the later ratio was not significant. Smaller mussels had relatively lower tissue N∶P ratio and higher condition factor. There was no difference in C∶P and N∶P ratios between seston and mussels' tissues. Our results indicated that the variation in nutrient stoichiometry of zebra mussels can be explained by food quality and quantity.

**Conclusions/Significance:**

Our study suggests that fitness of invasive zebra mussels is not constrained by nutrient stoichiometry which is likely to be important for their proliferation in novel ecosystems. The lack of imbalance in C∶P and N∶P ratios between seston and mussels along with high tissue C∶P ratio of the mussel allow them to tolerate potential P limitation and maintain high growth rate. Moreover, zebra mussels are able to change their tissue C∶P and N∶P ratios in response to the variation in elemental composition of their food. This can also help them to bypass potential nutrient stoichiometric constraints. Our finding is an important step towards understanding the mechanisms contributing to the success of exotic species from stoichiometric principles.

## Introduction

Biological invasion is an ubiquitous form of global change of biota, capable of causing native extinctions and affecting geographical speciation [1], [2] as well as the ecology and economy of the recipient ecosystems [3]–[7]. However, only a small fraction of the invaders can proliferate and/or spread in the invaded ecosystems [2]. The proximate causes of invasion success are not well understood [8], [9] and a major challenge in invasion ecology is to understand how a successful invader deals with ecological barriers in the novel ecosystems. For instance, changes in resource level or ratios may change the invasive potential of exotic species [10]. Measuring the mass balance of multiple key elements (C, N, and P), i.e. ecological stoichiometry [11], has emerged as a powerful tool for understanding mechanisms and processes important for the structure, biodiversity, and function of aquatic communities. Integrating the stoichiometric conceptual framework into the discipline of invasion biology can help us to address questions about how the structure of the invaded ecosystem interacts with the physiology of the invader to allow for the proliferation of the invader. This is critical to better understand the exotic species' population dynamics and extend the implication of biological invasion to the ecosystem level [12], [13].

At anthropogenic eutrophication, nutrient enrichment increases in the freshwater ecosystems. P enrichment may lead to increased algal C content [*but see* 14] more rapidly than algal P∶C ratio, thereby shifting the limiting factor from food quantity to quality for the grazer population, which in turn constrain the growth of grazers [12]. Such stoichiometric constrains may be described as a “paradox of enrichment” situation where increased nutrient input can destabilize the dynamics of consumers and may ultimately drive extinction or prevent invasion [12], [15], [16]. However, both resources quality, defined as the elemental imbalance of C∶N∶P ratios [17], [18], and resource quantity can limit consumer growth [11], [12] owning to tight coupling between growth rate, RNA allocation and biomass P content in consumers (the growth rate hypothesis GRH) [11], [19] and influence invasion success of alien species [15], [16]. Proliferation of an invasive species in a novel environment partly depends on its growth rate; high growth rates leads to high density and stronger ecological effects [20]. Thus, factors constraining the invader's growth rate limit its proliferation and invasion success. However, if exotic species can change their nutrient stoichiometry in response to nutrient composition of food available in the novel ecosystem while maintaining high growth, they may be very effective at exploiting resources and may have high capacity to expand. In fact, successful species tend to have wide environmental tolerance and high reproductive rates at all stages of the invasion process [4].

Although native species have shown to possess higher performance than exotic species when they are exposed to low resource conditions [21], [22], the zebra mussel (*Dreissena polymorpha*) has been shown to exploit available niche opportunities in the novel environment [23] and outperform natives [3], [5]. *Dreissena* has been one of the most successful invading species in European and North American lakes with rapid growth rate and flexible feeding behaviour capable of changing phytoplankton community structure, energy pathway, nutrient cycling, seston stoichiometry, and energy transfer efficiency from primary producers to upper trophic levels in both the pelagic and benthic food webs [3]–[7]. Zebra mussels feed on a wide size range of seston particles including phytoplankton [5], [6], [24], large bacteria [25], clay particles and detritus animals [24], protozoans, small zooplankton, and their own larvae [3], [26]. They have been documented to filter 53 tons of seston per hour in the Szczecin Gulf (Poland) and reduce the concentration of seston 2.3–6.9 times in Narochanskie lake system (Belarus) and threefold in Lukomskoe Lake (Belarus) [27].

We examined the relation between C∶P and N∶P ratios and the condition factor [(tissue condition index: TCI)] of zebra mussels collected from six sampling sites at each of two Swedish lakes: Lake Erken and Lake Ekoln. TCI is often used as a measure for the well being or health of mussels [28]. It has been shown that healthy marine mussels grow at a faster rate [29], [30] and that condition index can be a good indicator of growth in fan mussel (*Pinna bicolor*) [31], Mediterranean mussels (*Mytilus galloprovincialis*) [32], and blue mussels (*Mytilus edulis*) [30]. Zebra mussels with high TCI would have better physiological fitness and condition [33], [34], and would grow faster. Thus, based on GRH, we hypothesized that there is a negative correlation between condition factor and tissue N∶P and C∶P ratios of the mussels, which can constrain invasion success of zebra mussels. But to understand how the zebra mussel, as a successful invader, handle this stoichiometric constraints, we concurrently analyzed the elemental composition of the food (seston) and tissue of the mussels collected from a sampling site in Lake Erken in which nutrient composition of food and mussels varied over time. Our second hypothesis was that nutrient stoichiometry of zebra mussels is a function of elemental composition of their food and that there is no imbalance in C∶P and N∶P ratios between mussels' food and tissue. If so, zebra mussels might be able to cope with nutrient (phosphorus in particular) deficiencies in the novel ecosystems and bypass potential nutrient stoichiometric constraints. The current study represents an important step in understanding the potential importance of ecological stoichiometry to population dynamics and invasion success of a versatile invasive species such as the zebra mussel.

## Results

### Tissue C∶P and N∶P ratios and condition factor

**Figure 1:**
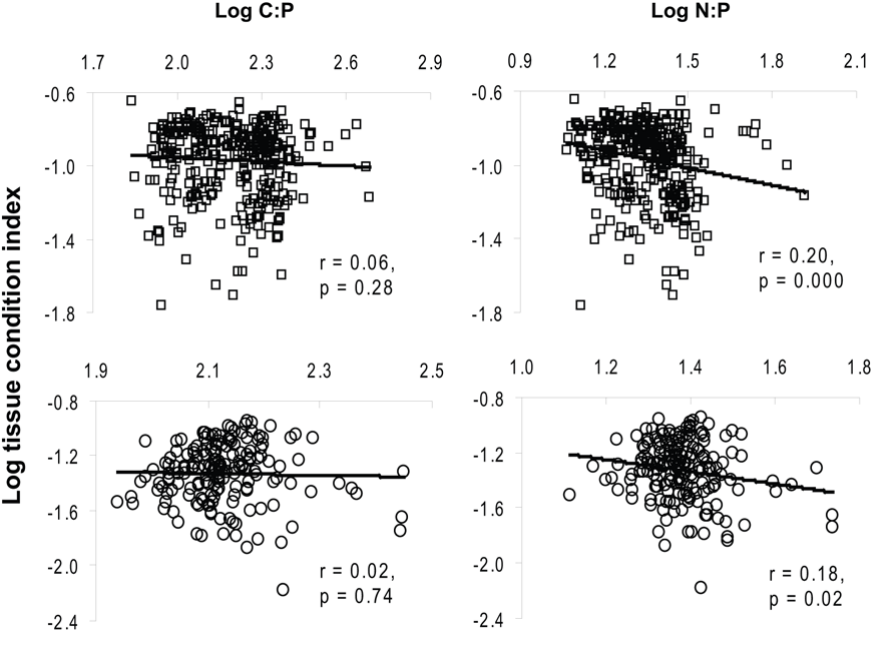
Regressions of Tissue Condition Index (TCI) on tissue stoichiometry (C∶P and N∶P molar ratios) of the zebra mussels collected from Lake Erken (top panels) and Lake Ekoln (bottom panels).

There was no significant relation between tissue C∶P molar ratio and TCI (p>0.05; Fig. 1), although TCI tended to decreases slightly as the tissue C∶P increased. A weak negative correlation between TCI and tissue N∶P molar ratio in both Lake Erken [Fig. 1; top panel, log(TCI) = −0.31 log (N∶P ratio)−0.54, *n* = 338] and Lake Ekoln [Fig. 1; bottom panel, log(TCI) = −0.44 log (N∶P ratio)−0.72, *n* = 178] was observed.

**Figure 2:**
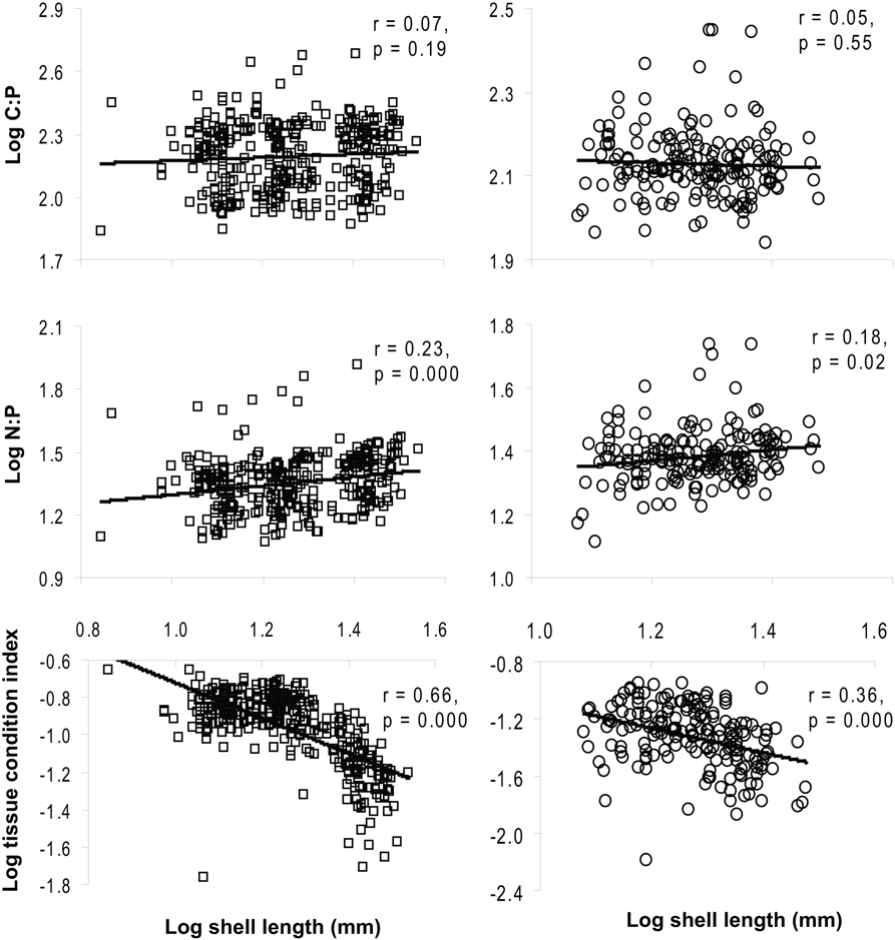
Regressions of tissue stoichiometry (C∶P and N∶P molar ratios) and Tissue Condition Index (TCI) of zebra mussels collected from Lake Erken (left panels) and Lake Ekoln (right panels), on shell length (mm) of the sampled mussels.

**Figure 3:**
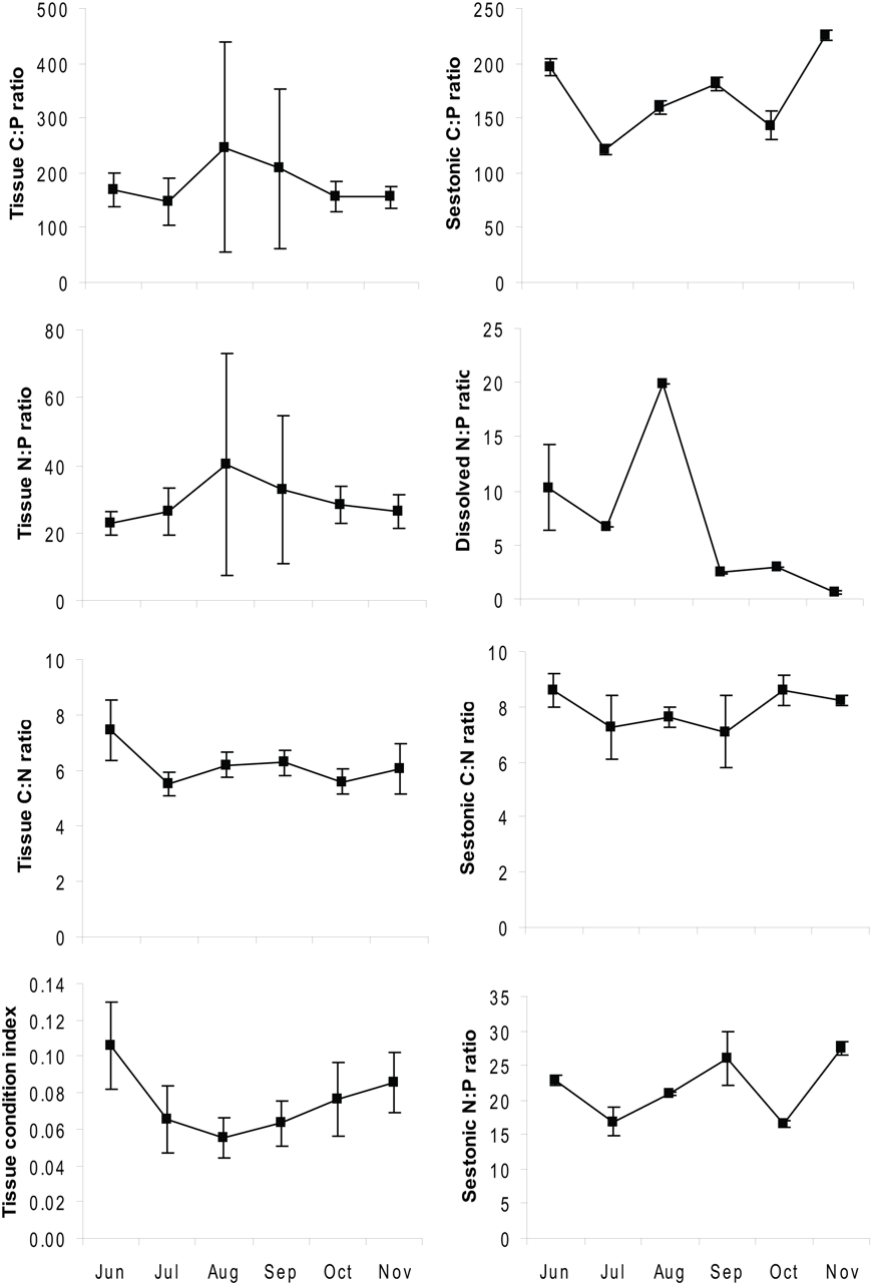
Changes in tissue and sestonic C∶N∶P ratio, dissolved N∶P ratio, and tissue condition index of zebra mussels in Lake Erken over time. Values represent means (±SD).

Size had no effect on tissue C∶P ratio (p>0.05; Fig. 2A), a relative positive effect on tissue N∶P ratio of the mussels from Lake Erken [Fig. 2; left panel, log(N∶P ratio) = 0.21 log (shell length, mm)+1.08, *n* = 338] and Lake Ekoln [Fig. 2; right panel, log(N∶P ratio) = 0.17 log (shell length, mm)+1.17, *n* = 178], and a negative effect on the TCI [Fig. 2; left panel, log(TCI) = −0.95 log (shell length, mm)+0.23, *n* = 338 for Lake Erken; and right panel, log(TCI) = −0.85 log (shell length, mm)−0.25, *n* = 178 for Lake Ekoln]. In Lake Erken, condition index of zebra mussels varied over time (χ^2^ = 117.4, df = 5, p<0.001, Fig. 3).

### Tissue C∶N∶P stoichiometry a function of seston nutrient composition

Tissue C∶P (χ^2^ = 23.3, df = 5, p<0.001), N∶P (χ^2^ = 34.8, df = 5, p<0.001), and C∶N (χ^2^ = 108.5, df = 5, p<0.001) ratios of *Dreissena* varied among months (Fig. 3). Nutrient content and stoichiometry of the food along with chlorophyll *a* (Chl *a*) explained the variation in tissue C∶N∶P stoichiometry of zebra mussels over time (Table 1). TP and Chl *a* concentrations had a negative effect on the elemental stoichiometry of the mussels (Table 1). There was a positive correlation between dissolved N∶P ratio and tissue N∶P ratio (Table 1, Fig. 3), sestonic C∶P ratio and tissue C∶N ratio, as well as tissue and sestonic C∶N ratio (Table 1, Fig. 3).

**Table 1:** Results of stepwise regression of tissue C∶N∶P stoichiometry of zebra mussel in Lake Erken.

Dependent variable	Independent variable	Slope	Constant	Model r
Tissue C∶P ratio	TP (µg L^−1^)	−3.05***	255.29***	0.23
Tissue N∶P ratio	Dissolved N∶P ratio	0.78***	97.02***	0.29
	Seston C∶N ratio	−8.4***		
	Chl *a* (µg L^−1^)	−4.4**		
Tissue C∶N ratio	TP (µg L^−1^)	−0.11***	3.95***	0.69
	Seston C∶N ratio	0.61***		
	Dissolved N∶P ratio	−0.06***		
	Seston C∶P ratio	−0.004*		

(n = 234 for each dependent variable).

* p<0.05.

** p<0.01.

*** p<0.001.

When the data from all months were combined, tissue C∶P, N∶P, and C∶N molar ratios of the mussels were (mean±1SD) 179.1±104.8, 29.3±17.1, and 6.2±0.9 in Lake Erken. Although the mean sestonic C∶P and N∶P molar ratios did not differ from those of the mussels, there were significant differences between the mussels and seston in their C∶N molar ratio (Table 2). However, coefficient of variation of C∶N∶P molar ratios of seston was almost equal to that of the mussels body tissues (Table 2).

**Table 2:** Comparison of descriptive statistics for molar ratios of C∶P, N∶P, and C∶N from zebra mussel and seston sampled from the sampling site at the eastern part of Lake Erken (site G) during June-November 2005.

Ratio		Zebra mussel	Seston	Paired samples T test	
				t	p-value
C∶P	n	6	6		
	Mean	180.2	171.3	0.4	0.7
	SD	38.7	37.8		
	CV (%)	21	22		
N∶P	n	6	6		
	Mean	29.4	21.8	2.5	0.06
	SD	6.2	4.5		
	CV (%)	21	21		
C∶N	n	6	6		
	Mean	6.2	7.9	−5.4	0.003
	SD	0.7	0.6		
	CV (%)	11	9		

## Discussion

The results presented in Fig. 1 partly supported the GRH hypothesis [11] and also our first hypothesis. Zebra mussel condition index was negatively related only to the their own tissue N∶P ratio but with a low correlation. Thus, zebra mussels' physiological fitness and successfulness in novel ecosystems are only partly affected by P. It has been demonstrated that growth rate, RNA allocation and biomass P content in biota are tightly coupled during ontogeny, and under physiological P limitation [19] and P enrichment in the food can lead to increased growth rate in zooplankton [35] and in some benthic invertebrates [36]. Furthermore, high values of tissue N∶P and C∶P ratios in August and September corresponded well with low condition index of zebra mussels, whereas *Dreissena* were in a relatively better condition in June and November when their tissue N∶P and C∶P ratio were relatively low (see Fig. 3.). However, the possibility that variation in TCI over time was affected by reproductive cycles cannot be excluded. Hence, it appears that there is a coupling, however not strong, between *Dreissena* fitness and tissue P content and probably food P concentrations.

Smaller mussels had relatively lower tissue N∶P ratio and better condition. Similarly, a small size-class of fan mussels has been observed to have higher condition index and growth rate than medium and larger size-class mussels [31]. Hickman and Illingworth (1980) also found an inverse relationship between condition index and size of green-lipped mussels (*Perna canaliculus*). Smaller *Dreissena*, therefore, would presumably grow faster than large *Driessena* [37]. The positive relation between N∶P ratio and body size among invertebrates is due to the negative allometric scaling of growth rate to body size [11]. Increasing P content along with higher growth rate with decreasing body size is a pattern that has been observed in littoral mayflies [36], pelagic invertebrates [38], [39] and many benthic invertebrates [40]. Still, if food quality is low, ontogenetic shifts in stoichiometric constraints could result in a stoichiometric bottleneck in fast-growing juveniles [41], which have elevated P levels and demands. Zebra mussels appear to devote a larger proportion of their body tissue and more energy to reproduction than to growth [42], suggesting strong effects of stoichiometric imbalance on reproductive output [41]. Thus small mussels with good condition index and high growth rate require food with high P content to maintain optimal growth and reproduction and are therefore, more likely to suffer from reduced food quality. Moreover, higher respiration rates for small mussels can translate into higher energetic costs and lower energy available for investment in growth or reproduction, considering that respiration alone can account for 88% of the caloric intake [43]. Previous studies documented that reproduction of herbivorous zooplankton was reduced as a result of high C: nutrient ratios in algae [44] and reproductive effort and egg mass of zebra mussels increased when they were provided with low lipid diet [45] and high quality phytoplankton, respectively [46]. Consequently, when a large proportion of the population consist of small individuals, the mussel's reproduction and proliferation in the colonized habitats may be affected by poor food quality. Nevertheless, the carrying capacity or equilibrium density of populations should decline with increasing body size [47].

A relevant question in relation to the discussion above is how zebra mussels are successful in a novel ecosystem despite stoichiometric constraints. Unlike native mussels, zebra mussels are *r* strategists and have rapid growth rate [48], but they also have lower P content (0.9% of soft tissue; this study) than native mussels (e.g., *Lampsilis radiata siliquoidea*, 2.4–3.4%; [49]). This is presumably a result of the lower P requirements of zebra mussels (see below). A recent study implied that although exotic *Daphnia lumholtzi* has a growth rate, P content, and fecundity similar to their native congeners *D. magna* and *D. pulicaria*, it contains a higher RNA level which may facilitate quicker resting egg production resulting in higher chance of invasion success [50]. Future investigation might determine whether there is a relation between RNA allocation, growth rate and body P content of the mussels.

In fact, the high value (mean and standard deviation) of tissue C∶P ratio in the zebra mussels in this study, is higher than that for most of the benthic invertebrates in the littoral zone of Lake Erken [40]. Thus, *Dreissena* can be surmised to have low P demands and thus be less sensitive to P deficiency. According to resource-ratio theory, the species that survives at the lowest levels of a limiting resource will be the superior competitor for that resource [15], [51], indicating that invasive *Dreissena* could be successful in P-limited environments and become very effective at exploiting resources. Further, experiments with dietary P supplements are needed to address whether high tissue C∶P ratio of the zebra mussel is a result of consumption of food having high C∶P ratio and whether the mussels' growth rate is affected by such food. Based on physiological models, when herbivores are exposed to a value above critical C∶P levels of food, they maintain their homeostasis in C∶P ratio, which can lead to a reduction in their growth rate [52]. For example, when *Daphnia* fed on high C∶P ratio food, a direct P deficiency in this herbivore was indicated [35]. But a previous study also demonstrated a higher range of variability in C∶P ratio of *Dreissena* than that of invertebrates such as snails (*Theodoxus fluviatilis*) and tube-making caddisflies (Polycentropodidae) among seasons [40], suggesting that zebra mussels are more flexible in their tissue C∶P ratio. Alternatively, high C∶P ratio of the mussels' tissues could be caused by a decrease in soft tissue P content during the summer as a result of gametogenesis, as has been observed in zebra mussels in western Lake Erie [53]. In addition, during the period of our study, the mussels had probably a lower protein content due to gamete production and higher carbohydrate, which are high in C and low in P [11], thus correspondingly higher C∶P ratio.

Our results also revealed an equal coefficient of variation of C∶N∶P ratio and lack of imbalance in C∶P and N∶P ratios between seston and mussels, suggesting that *Dreissena* are not restricted in the range of their body mass stoichiometry and may not be P limited if they graze on seston (supporting our second hypothesis). However, zebra mussels similar to other invertebrates in Lake Erken had smaller C∶N ratio than their food [40]. Such smaller C∶N ratio can be a result of lower C content in consumers than their plant prey [11].

Further, we indicated that the variation in nutrient stoichiometry of zebra mussels can partly be explained by productivity, food quality in term of C∶N and C∶P ratios, food quantity in term of Chl *a* concentrations, and dissolved N∶P ratio of the water (supporting our second hypothesis). This suggests that consumers' stoichiometry can be affected by dietary changes through elemental stoichiometry, corroborating findings from both pelagic [35] and benthic [36] food webs. A positive correlation was observed between tissue N∶P ratio and dissolved N∶P ratio as well as tissue and sestonic C∶N ratio (Table 1). Similarly, when the dissolved inorganic N∶P ratio of seston and periphyton N∶P ratio were low in Lake Erken in autumn, the invertebrate N∶P ratios were also low [54], [55]. It has also been revealed that zooplankton C∶P ratio is strongly correlated to the sestonic C∶P ratio [56]. Thus, zebra mussels are able to change their tissue nutrient stoichiometry in response to the variation in elemental composition of their food. This can in turn assist them to bypass the stoichiometric constraints of their food resources. However, in addition to food stoichiometry, seasonal differences in growth and reproduction can lead to the seasonal variation in nutrient contents of the consumers [11].

It has been shown that TP is negatively correlated with the C∶P ratio of phytoplankton [57] and seston [52]. Because zebra mussel are actively feeding on phytoplankton [6], their tissue C∶P ratio can be related to the phytoplankton C∶P ratio, a relation observed in zooplankton [56]. This may explain why TP has a negative effect on zebra mussel tissue C∶P ratio. In addition, *Dreissena* are stripping the P from the water column through feeding on Chl *a* [7]. The mussel's ingestion rate increases linearly with food concentration (Chl *a*) until an asymptote is reached at 2 g C L^−1^ [58]. Considering that such levels were not observed in our study, it is likely that the mussels ingest more phytoplankton as the Chl *a* increases in the water, thereby sequester more phosphorous in their tissue which in turn can lead to a reduction in their N∶P ratio. This could possibly explain the negative effect of Chl *a* on the tissue N∶P ratio. On the other hand, because increasing Chl *a* leads to an increase in the mussels' growth rate [59], which is negatively related to the tissue N∶P ratio (this study), it seems reasonable to expect a negative relation between Chl *a* and tissue N∶P ratio. The concentration of Chl *a* was also proposed to be an important factor in the elemental composition of a calanoid copepod, *Mixodiaptomus laciniatus* [38]. Hence, where there is a food (Chl *a*) limitation, we can expect an increase in the tissue N∶P ratio, a reduction in the growth rate and lower invasion success of the zebra mussels.

Taken together, this study suggests that nutrient stoichiometry cannot reduce the fitness of invasive zebra mussel, and thus, is not able to constrain their proliferation in novel ecosystems. The lack of imbalance in C∶P and N∶P ratios between seston (food) and mussels along with high tissue C∶P ratio of the mussel allow them to tolerate potential P limitation, and thus, maintain high growth rate and proliferate in many novel ecosystems. Moreover, zebra mussels are able to change their tissue C∶P and N∶P ratios in response to the variation in elemental composition of their food. This can also help them to bypass potential nutrient stoichiometric constraints. Further, zebra mussel ability to exploit dissolved organic carbon [60], a main carbon pool in lakes, may explain somewhat the invasion success of this species. We anticipate our finding to be an important step towards understanding the mechanisms contributing to the success of exotic species by explaining invasion success from stoichiometric principles. These findings can improve our ability to predict which introduced species becomes a successful invader.

## Materials and Methods

### Study area

The study was conducted in two Swedish lakes. Lake Erken which is a mesotrophic (Mean total phosphorus (TP), 27 µg L^−1^) lake with a relatively small catchment dominated by forest, and has a surface area of approximately 24 km^2^, a mean depth of 9 m, and a maximum depth of 21 m and Lake Ekoln which is a sub-basin of Lake Mälaren, a rather deep eutrophic lake (Mean TP, 52 µg L^−1^) with a catchment more dominated by agriculture, and has a surface area of approximately 30 km^2^, a mean depth of 16 m, and a maximum depth of 50 m [61]. Zebra mussels invaded Lake Erken in mid-1975 [7] and Lake Malaren in 1926 [62]. An increase in water clarity and a reduction in Chl *a* concentration were immediately observed since zebra mussel establishment in Lake Erken (Erken database). Moreover, the occurrence of cyanobacteria, *Gloeotrichia echinulata*, blooms increased after zebra mussel invasion [7].

### Tissue C∶P and N∶P ratios and condition factor

#### Mussel sampling

To evaluate the relation between tissue C∶P and N∶P ratios and condition factor of the mussels, six sampling sites (sites A-F) were selected around each of the lake. Six transect lines were established perpendicular to the shoreline at all sites (one transect line per site) in Lake Erken in June 2005 and in Lake Ekoln in October 2006. Mussel samples were collected at each site by SCUBA diving, placed in buckets filled with lake water and transported to the laboratory [5]–[7]. In the laboratory, we detached the mussels from their natural substrate by severing their byssal threads with a scalpel. To determine mussels' body tissue nutrient content and condition factor, we first selected 40–60 individuals (7–34.7 mm shell length) per site from Lake Erken and 22–32 individuals (12–30 mm shell length) per site from Lake Ekoln and then brushed them clean to remove silt and algae adhering to the shells and placed the mussels overnight in water without food to allow them to empty their guts [7], [40]. However, a previous study showed a low variation of tissue C∶N∶P stoichiometry among mussels collected from one sampling site; suggesting a number of 15 individuals of mussels would provide a reasonable value of C∶N∶P ratio for mussels stoichiometry [7]. Individuals of each site were then placed separately in plastic tissue-culture plates and stored in the freezer for C, N, and P analysis and condition factor measurement.

#### Analysis of the mussels

Mussels were freeze dried to constant weight and the dry mass (DM) of each individual was determined [40]. After weighting, individual mussel shell length was measured, the soft tissue was scraped off from shells using a scalpel. Sample masses of 14 to 5510 µg were used for the analysis depending on mussel size. Similar tissue parts were sampled for all specimens and analyzed for C, N, and P tissue content. C and N contents of tissues were measured simultaneously with a CHN analyzer (LECO CHN-932, Carlo–Erba Strumentazione), and P content of tissues was measured as phosphate after hot hydrolysis with potassium persulfate [63]. The same individual was used for the P analysis as for the CHN analysis. C∶N, C∶P, and N∶P ratios were calculated in molar units. We determined condition factor of the mussels based on Tissue Condition Index (TCI = the tissue dry weight/shell dry weight) [28], [33]. Because we were interested in evaluating how tissue C∶N∶P ratio of mussels related to their fitness and successfulness, we calculated the condition index. Asymmetric growth of the shells is not a good indicator of “general health” of the zebra mussels [34].

### Tissue C∶N∶P stoichiometry and seston nutrient composition

#### Sampling

To address how mussel tissue C∶N∶P stoichiometry vary in relation to seston nutrient composition and Chl *a*, we performed a monthly sampling from the mussels and the water from the one sampling site at the eastern part of the Lake Erken during June-November 2005. Concurrent to the monthly collection of 38–40 mussels (8–35.5 mm shell length), we took water samples to measure the quantity (Chl *a*, particulate C, particulate P, and particulate N) and quality of food (seston C∶N∶P stoichiometry) available to zebra mussels and nutrient concentrations (total P, total N, ammonium-N, phosphate-P). The mussels were cleaned, kept overnight in water without food, and stored in the freezer. Three 2-l samples were taken about 25 cm above the lake bed at the sampling site. The water sample was divided into five different subsamples for each site. (1) for the analysis of TP and total nitrogen (TN); (2) to measure phytoplankton biomass (defined as Chl *a*); (3) for the analysis of ammonium-N (NH_4_^+^- N), nitrate- nitrite- nitrogen (NO_2_^−^NO_3_^−^- N), and phosphate-P (PO_4_^3−^-P); (4) water was filtered on precombusted GF/C filters for the analysis of particulate C and particulate N; (5) water was filtered on precombusted GF/C filters for the analysis of particulate P.

#### Mussels and water analyses

C, N, and P contents of the mussels' tissues were measured and the stoichiometric C∶N∶P ratio were calculated in molar units. Particulate C and particulate N of seston were measured using the same methods as for tissue nutrient analyses. Dissolved ammonium was analyzed with the Tecators method and total phosphorus and dissolved phosphate with the ammonium-molybdate method [63], in an auto-analyzer (Flow Injection Analyzer). In addition, molar ratios of C∶P, N∶P, C∶N, and dissolved N∶P (NH_4_∶PO_4_) for seston stoichiometry were calculated for each of the six sampling sites. Concentration of Chl *a* was determined using delayed fluorescence excitation spectroscopy [5]–[7].

#### Correlation

A Pearson correlation coefficient was performed to test for correlations between environmental variables. When the variables were collinear (p<0.05), only one variable was chosen. There was a high correlation between Chl *a*, PC, PP, PN, NO_2_^−^NO_3_^−^-N, and PO_4_^3−^-P (all r>0.82) as well as between setston C∶P ratio and N∶P ratio (r = 0.97). Chl *a* was preferred to other related parameters due to its importance in mussels growth [60] and its role as a main food resource for the mussels [7]. We assumed that sestonic C: nutrient ratio was more important than N∶P ratio because of its effect on herbivore growth [36], [64], [65]. Thus we included sestonic C∶P ratio and C∶N ratio in our model. Moreover, the independent variables such as TP, TN, dissolved inorganic nitrogen including NH_4_^+^- N, and dissolved N∶P ratio were considered in our model (*see* below) due to their role in growth of phytoplankton a food of herbivore mussels.

### Statistical analysis

The relationship between condition factor and tissue C∶P and N∶P ratios of the mussels was analyzed with linear regression. Linear regression was also applied to analyze the relationships between mussels size (shell length) against tissue C∶P and N∶P ratios and condition of zebra mussels. We performed stepwise multiple regressions to determine the factors that best predicated zebra mussel tissue C∶N∶P stoichiometry. Thus, we regressed separately the tissue C∶P, N∶P, and C∶N molar ratios of *Dreissena* against seston nutrient concentration (PN, TN, NH_4_^+^- N), dissolved N∶P ratio, sestonic C∶P and C∶N molar ratios, and Chl *a*. We used the paired samples T test to compare C∶N∶P stoichiometry between mussels tissue and seston. Data were log-transformed when necessary to meet assumptions of normality and homogeneity of variances. We used a non-parametric Kruskal Wallis to compare zebra mussel condition factor and tissue C∶N∶P stoichiometry among months. All statistical tests were performed with software package SPSS 16.0. Statistical significance was accepted at the *p*<0.05 level.

## References

[1] Cox GW (2004). Alien Species and Evolution.

[2] Kolar C, Lodge DM (2001). Progress in invasion biology: predicting invaders.. Trends Ecol Evol.

[3] MacIsaac HJ (1996). Potential abiotic and biotic impacts of zebra mussels on the inland waters of North America.. Am Zool.

[4] Côté LM, Reynolds JD (2002). Predictive ecology to the rescue?. Science.

[5] Naddafi R, Eklöv P, Pettersson K (2007). Non-lethal predator effects on the feeding rate and prey selection of the exotic zebra mussel *Dreissena polymorpha*.. Oikos.

[6] Naddafi R, Pettersson K, Eklöv P (2007). The effect of seasonal variation in selective feeding by zebra mussels (*Dreissena polymorpha*) on phytoplankton community composition.. Freshwater Biol.

[7] Naddafi R, Pettersson K, Eklöv P (2008). Effects of the zebra mussel, an exotic freshwater species, on seston stoichiometry.. Limnol Oceanogr.

[8] Holway DA, Suarez AV, Case TJ (1998). Loss of intraspecific aggression in the success of a widespread invasive social insect.. Science.

[9] Peacor SD, Allesina S, Riolo RL, Pascual M (2006). Phenotypic plasticity opposes species invasions by altering fitness surface.. PLoS Biol.

[10] Seimann E, Rogers WE (2007). The role of soil resources in an exotic tree invasion in Texas coastal prairie.. J Ecology.

[11] Sterner RW, Elser JJ (2002). Ecological stoichiometry.

[12] Moe SJ, Stelzer RS, Forman MR, Harpole WS, Daufresne T (2005). Recent advances in ecological stoichiometry: insights for population and community ecology.. Oikos.

[13] Ptacnik R, Jenerette GD, Verschoor AM, Huberty AF, Solimini AG (2005). Applications of ecological stoichiometry for sustainable acquisition of ecosystem services.. Oikos.

[14] Sterner RW, Andersen T, Elser JJ, Hessen DO, Hood J (2008). Scale-dependent carbon:nitrogen:phosphorus seston stoichiometry in marine and freshwaters.. Limnol Oceanogr.

[15] Tilman D (1982). Resource Competition and Community Structure.

[16] Lennon JT, Smith VH, Dzialowski AR (2003). Invasibility of plankton food webs along a trophic state gradient.. Oikos.

[17] Elser JJ, Fagan WF, Denno RF, Dobberfuhl D, Folarin A (2000). Nutritional constraints in terrestrial and freshwater food webs.. Nature.

[18] Mitra A, Flynn KJ (2007). Importance of interactions between food quality, quantity, and gut transit time on consumer feeding, growth, and trophic dynamics.. Am Nat.

[19] Elser JJ, Acharya K, Kyle M, Cotner J, Makino W (2003). Growth rate–Stoichiometry couplings in diverse biota.. Ecol Lett.

[20] Williamson M, Fitter A (1996). The varying success of invaders.. Ecology.

[21] Daehler CC (2003). Performance comparisons of co-occurring native and alien invasive plants: Implications for conservation and restoration.. Ann Rev Ecol Evol Syst.

[22] Seabloom EW, Harpole WS, Reichman OJ, Tilman D (2003). Invasion, competitive dominance, and resource use by exotic and native California grassland species.. Proc Natl Acad Sci.

[23] Shea K, Chesson P (2002). Community ecology theory as a framework for biological invasions.. Trends Ecol Evol.

[24] Baker SM, Levinton JS, Kurdziel JP, Shumway SE (1998). Selective feeding and biodeposition by zebra mussels and their relation to changes in phytoplankton composition and seston load.. J Shell Res.

[25] Cotner JB, Gardner WS, Johnson JR, Sada RH, Cavaletto JF (1995). Effects of zebra mussels (*Dreissena polymorpha*) on bacterioplankton: Evidence for both size-selective consumption and growth stimulation.. J Great Lakes Res.

[26] MacIsaac HJ, Sprules WG, Leach JH (1991). Ingestion of small-bodied zooplankton by zebra mussels (*Dreissena polymorpha*): can cannibalism on larvae influence population dynamics?. Can J Fish Aquat Sci.

[27] Karatayev AY, Burlakova LE, Padilla DK (1997). The effects of *Dreissena polymorpha* (Pallas) invasion on aquatic communities in eastern Europe.. J Shellfish Res.

[28] Soto M, Ireland MP, Marigomez I (2000). Changes in mussel biometry on exposure to metals: implictions in estimation of metal bioavailability in ‘Mussel-Watch’ programmes.. Sci Tot Environ.

[29] Nix ER, Fisher CR, Vodenichar J, Scott KM (1995). Physiological ecology of a mussel with methanotrophic endosymbionts at three hydrocarbon seep sites in the Gulf of Mexico.. Mar Biol.

[30] Smaal AC, Stralen MRV (1990). Average annual growth and condition of mussels as a function of food source.. Hydrobiologia.

[31] Wu RSS, Shin PKS (1998). Transplant experiments on growth and mortality of the fan mussel *Pinna bicolor*.. Aquaculture.

[32] Thébault H, Arnaud M, Charmasson S, Andral B, Diméglio Y (2005). Bioavailability of anthropogenic radionuclides *in* mussels along the French Mediterranean coast.. Radioprotection.

[33] Smolders R, Bervoets L, De Coen W, Blust R (2004). Cellular energy allocation in zebra mussels exposed along a pollution gradient: linking cellular effects to higher levels of biological organization.. Environ Poll.

[34] Voets J, Talloen W, de Tender T, van Dongen S, Covaci A (2006). Microcontaminant accumulation, physiological condition and bilateral asymmetry in zebra mussels (*Dreissena polymorpha*) from clean and contaminated surface waters.. Aquat Toxicol.

[35] Urabe J, Clasen J, Sterner RW (1997). Phosphorus-limitation of *Daphnia* growth: Is it real?. Limnol Oceanogr.

[36] Frost PC, Elser JJ (2002). Growth responses of littoral mayflies to the phosphorus content of their food.. Ecol Lett.

[37] Hickman RW, Illingworth J (1980). Condition cycle of the green-lipped mussel Perna canaliculus in New Zealand.. Mar Biol.

[38] Villar-Argaiz M, Medina-Sánchez JM, Carrillo P (2002). Linking life history strategies and ontogeny in crustacean zooplankton: implications for homeostasis.. Ecology.

[39] Vrede T, Dobberfuhl DR, Kooijman SALM, Elser JJ (2004). Fundamental connections among organism C∶N∶P stoichiometry, macromolecular composition, and growth : Stoichiometric Ecology.. Ecology.

[40] Leiss A, Hillebrand H (2005). Stoichiometric variation in C∶N, C∶P and N∶P ratios of littoral benthic invertebrates.. J N Am Benthol Soc.

[41] Andersen T, Elser JJ, Hessen DO (2004). Stoichiometry and population dynamics.. Ecol Lett.

[42] Stoeckmann A (2003). Physiological energetics of Lake Erie dreissenid mussels: a basis for the displacement of *Dreissena polymorpha* by *Dreissena bugensis*.. Can J Fish Aquat Sci.

[43] Stoeckmann AM, Garton DW (1997). A seasonal energy budget for zebra mussels (*Dreissena polymorpha*) in western Lake Erie.. Can J Fish aquat Sci.

[44] Sterner RW, Hessen DO (1994). Algal nutrient limitation and the nutrition of aquatic herbivores.. Annu Rev Ecol Syst.

[45] Stoeckmann AM, Garton DW (1994). Energy allocation strategies of the zebra mussel under differing environmental conditions.. Amer Zool.

[46] Wacker A, von Elert E (2003). Food quality controls reproduction of the zebra mussel (*Dreissena polymorpha*).. Oecologia.

[47] Savage VM, Gillooly JF, Brown JH, West GB, Charnov EL (2004). Effects of body size and temperature on population growth.. Am Nat.

[48] Vanderploeg HA, Nalepa TF, Jude DJ, Mills EL, Holeck KT (2002). Dispersal and emerging ecological impacts of Ponto-Caspian species in the Laurentian Great Lakes.. Can J Fish Aquat Sci.

[49] Nalepa TF, Gardner WS, Malczyk JM (1991). Phosphorus cycling by mussels (Unionidae: Bivalvia) in. Lake St. Clair.. Hydrobiologia.

[50] Acharya K, Jack JD, Smith AS (2006). Stoichiometry of *Daphnia lumholtzi* and their invasion success: Are they linked?. Arch Hydrobiol.

[51] Miller TE, Burns JH, Munguia P, Walters EL, Kneitel JM (2005). A critical review of twenty years' use of the resource-ratio theory.. Am Nat.

[52] Sterner RW, Fee EJ, Guildford SJ, Chrzanowski TH (1997). The light: nutrient ratio in lakes: The balance of energy and materials affects ecosystem structure and process.. Am Nat.

[53] Arnott DL, Vanni MJ (1996). Nitrogen and phosphorus recycling by the zebra mussel (*Dreissena polymorpha*) in the western basin of Lake Erie.. Can J Fish Aquat Sci.

[54] Hillebrand H, Kahlert M (2001). Effect of grazing and nutrient supply on periphyton biomass and nutrient stoichiometry in habitats of different productivity.. Limnol Oceanogr.

[55] Kahlert M, Hasselrot AT, Hillebrand H, Pettersson K (2002). Spatial and temporal variation in the biomass and nutrient status of epilithic algae in Lake Erken, Sweden.. Freshwater Biol.

[56] Hessen DO, Van Donk E, Gulati R (2005). Seasonal seston stoichiometry: effects on zooplankton in cyanobacteria dominated lakes.. J Plankton Res.

[57] Qin P, Mayer CM, Schulz KL, Ji X, Ritchie M (2007). Ecological stoichiometry in benthic food webs: Effect of light and nutrients on periphyton food quantity and quality in lakes.. Limnol Oceanogr.

[58] Walz N (1978). The energy balance of the freshwater mussel *Dreissena polymorpha* (Pallas) in laboratory experiments and in Lake Constance: I. Pattern of activity, feeding and assimilation efficiency.. Arch Hydrobiol.

[59] Jantz B, Neumann D (1998). Growth and reproductive cycle of the zebra mussel in the River Rhine as studied in a river bypass.. Oecologia.

[60] Roditi HA, Fisher NS, Sañudo-Wilhelmy SA (1998). Uptake of dissolved organic carbon and trace metals by zebra mussels.. Nature.

[61] Malmaeus JM, Blenckner T, Markensten H, Persson I (2006). Lake phosphorus dynamics and climate warming: A mechanistic model approach.. Ecol Model.

[62] Josefsson M, Andersson B (2001). The environmental consequences of alien species in the Swedish lakes Mälaren, Hjälmaren, Vänern and Vättern.. Ambio.

[63] Grasshoff K, Ehrhardt M, Kremling K (1983). Methods of seawater analysis.

[64] White TCR (1993). The inadequate environment: nitrogen and the abundance of animals.

[65] Hessen DO, Færøvig P, Andersen T (2002). Light, nutrients, and P∶C ratios in algae: grazer performance related to food quality and quantity.. Ecology.

